# The Storage Period Discrimination of Bolete Mushrooms Based on Deep Learning Methods Combined With Two-Dimensional Correlation Spectroscopy and Integrative Two-Dimensional Correlation Spectroscopy

**DOI:** 10.3389/fmicb.2021.771428

**Published:** 2021-11-25

**Authors:** Jian-E Dong, Ji Zhang, Tao Li, Yuan-Zhong Wang

**Affiliations:** ^1^College of Big Data and Intelligence Engineering, Southwest Forestry University, Kunming, China; ^2^Medicinal Plants Research Institute, Yunnan Academy of Agricultural Sciences, Kunming, China; ^3^College of Chemistry, Biological and Environment, Yuxi Normal University, Yuxi, China

**Keywords:** bolete, two-dimensional correlation spectroscopy (2DCOS), deep learning, residual convolutional neural network (ResNet), storage period

## Abstract

Boletes are favored by consumers because of their delicious taste and high nutritional value. However, as the storage period increases, their fruiting bodies will grow microorganisms and produce substances harmful to the human body. Therefore, we need to identify the storage period of boletes to ensure their quality. In this article, two-dimensional correlation spectroscopy (2DCOS) images are directly used for deep learning modeling, and the complex spectral data analysis process is transformed into a simple digital image processing problem. We collected 2,018 samples of boletes. After laboratory cleaning, drying, grinding, and tablet compression, their Fourier transform mid-infrared (FT-MIR) spectroscopy data were obtained. Then, we acquired 18,162 spectral images belonging to nine datasets which are synchronous 2DCOS, asynchronous 2DCOS, and integrative 2DCOS (i2DCOS) spectra of 1,750–400, 1,450–1,000, and 1,150–1,000 cm^–1^ bands. For these data sets, we established nine deep residual convolutional neural network (ResNet) models to identify the storage period of boletes. The result shows that the accuracy with the train set, test set, and external validation set of the synchronous 2DCOS model on the 1,750–400-cm^–1^ band is 100%, and the loss value is close to zero, so this model is the best. The synchronous 2DCOS model on the 1,150–1,000-cm^–1^ band comes next, and these two models have high accuracy and generalization ability which can be used to identify the storage period of boletes. The results have certain practical application value and provide a scientific basis for the quality control and market management of bolete mushrooms. In conclusion, our method is novel and extends the application of deep learning in the food field. At the same time, it can be applied to other fields such as agriculture and herbal medicine.

## Introduction

Wild edible mushrooms are favored by consumers for their rich nutrients, unique flavor, and special food and medicinal values. They are rich in protein, vitamins, mineral elements, dietary cellulose, and other nutrients, as well as polysaccharides, nucleosides, alkaloids, and other functional ingredients (Sande et al., 2019; Dong et al., 2021a; Wang et al., 2021). Long-term consumption of wild edible mushrooms can enhance body immunity, delay aging, and regulate human metabolism (Xu, 2019). One study found that the output of edible mushrooms in the world increased by about 30 times from 1978 to 2013, of which China is the major producer of edible mushrooms, accounting for 85% of the world production (Mleczek et al., 2018). From 2009 to 2013, the export value of China’s edible mushrooms increased 142 times (Li et al., 2016). In 2018, the national output of edible mushrooms reached 38,420,000 tons, and the export volume was $4.458 billion (He, 2019; Wang, 2020). Due to the diversity of its geography and climate, Yunnan Province provides a good environment for the growth of wild edible mushrooms (Dong et al., 2021b). In Yunnan, there are more than 900 species of wild edible mushrooms, which account for about 50% of the world’s resources and over 90% of China’s domestic resources (Mary et al., 2015). Bolete is a kind of precious wild edible mushroom with thick meat, delicious taste, and unique aroma. Moreover, bolete is rich in proteins, amino acids, carbohydrates, and total sterol, with low lipid content and energy value, so it is favored by consumers (Chen et al., 2021). There are 397 species of boletes belonging to 28 genera in China, of which 199 are edible species (Yang T. W. et al., 2014). Yunnan is rich in bolete resources, and it is one of the regions with the largest species and quantities of boletes in the world (Sarikurkcu et al., 2015). In addition, there are 224 known species of boletes in Yunnan, which account for 56.4% of the resources of boletes in China (Yang T. W. et al., 2014).

The growth of wild-grown boletes is restricted by season, and their harvest season is from June to September each year. Fresh boletes are perishable and easily damaged during transportation. At the same time, in order to meet the market demand to ensure a year-round supply, boletes are mostly dried and dehydrated, and then they are made into a durable storage commodity which is easy to be transported (Chen and Xu, 2013). The processing and treatment of wild-grown boletes are often not sterilized. With the increase of storage period, the internal microorganisms will proliferate and produce amines, mercaptans, hydrogen sulfide, indoles, and other compounds, which will cause harm to human health (Zhang et al., 2018). Flavor and aromatic substances are very important for food, and volatile substances are the key factors affecting the flavor of wild-grown boletes. Due to the high moisture content of the fruity body of fresh boletes, most of them are processed into dry pieces for preservation and sale, which leads to the frequent occurrence of the phenomenon of false for true, and it is difficult to carry out quality control and market management (Yang T. W. et al., 2016). Therefore, the identification of the storage period of boletes has great significance to ensure its quality.

At present, Fourier transform infrared (FTIR) spectroscopy is widely used in the research field of Chinese herbal medicine and food identification (Bassbasi et al., 2014; Yang Y. et al., 2016). This technology can be used for rapid, simple, and nondestructive detection of samples, and it can effectively reflect the composition characteristics of complex substances. However, the ingredients of the samples are often very complex, which results in the overlap of the spectral signals, so it is difficult to extract useful information from the FTIR spectral signals (Chen et al., 2018). 2DCOS is a versatile tool which can extract useful information under some chemical or physical stimulus from a series of spectra. We can get the synchronous and asynchronous correlation spectra according to the cross-correlation analysis of spectral variations caused by perturbation. Moreover, 2DCOS has higher spectral resolution. The characteristic information of weak peak, migrating peak, and overlapping peak can be extracted effectively. Therefore, it is a beneficial alternative to standard infrared spectroscopy (Noda, 2014a,b, 2016, 2018; Yang et al., 2020). In recent years, 2DCOS has been widely used in the field of Chinese herbal medicine and food identification. Walkowiak et al. (2019) detected the adulterants in dietary supplements with Ginkgo biloba extract by 2DCOS. Qu et al. (2016) used 2DCOS for chemical profiling and adulteration screening of Aquilariae Lignum Resinatum. Chen et al. (2018) adopted i2DCOS technology to visually identify the adulteration of Chinese medicinal materials. Noda (2018) studied the D-glucose anomers undergoing a mutarotation process in water by 2DCOS.

Nowadays, computer processing methods are widely used in chemical data analysis. When traditional machine learning methods are used to identify 2DCOS spectral data, there is a large amount of data and the processing process is complex, which requires artificial design and extraction of characteristic variables. Deep learning is excellent at processing high-dimensional data with complex structures. It is currently used in image recognition, speech recognition, drug molecular activity prediction, particle accelerator data analysis, prediction of the effect of DNA mutations on gene expression, agricultural production, and natural language understanding (Guo et al., 2015; LeCun et al., 2015; Kamilaris and Prenafeta-Boldú, 2018). A convolutional neural network (CNN) is an efficient recognition method developed in recent years and has attracted widespread attention. It has been successfully applied to image recognition (Albawi et al., 2017). Generally, the processing effect is better with the depth of network increase in CNN. However, there is a gradient explosion problem in the deep network, which leads to the model accuracy degradation (He et al., 2016; Jiao et al., 2017). Gradient explosion refers to the continuous accumulation of large error gradients during neural network training, which results in significant updating of model weights. It will cause the model to be unstable and unable to use the training data to learn. The residual convolutional neural network (ResNet) can overcome the problem of gradient explosion caused by network deepening of CNN (Wu et al., 2019). At present, the application of deep learning in the identification field of boletes is rare, and the identification of food and Chinese medicinal materials using the ResNet method combined with 2DCOS is even less. In this article, ResNet combined with 2DCOS was used to identify the storage period of wild-grown boletes, which is an innovation in this field.

In this research, we collected 2,018 fruiting bodies of wild-grown boletes for four storage periods in Southwest China (mainly in Yunnan Province). Then we intercepted the 1,750– 400-, 1,450–1,000-, 1,150–1,000-cm^–1^ bands and used the two-trace two-dimensional (2T2D) (Yang et al., 2020) method to generate the synchronous 2DCOS, asynchronous 2DCOS, and i2DCOS spectra of all samples. In this way, nine data sets and a total of 18,162 2DCOS spectral images were obtained. Finally, we established ResNet models for these nine data sets and analyzed the models through accuracy, loss values, and external verification results to find the optimal model. Therefore, the deep learning method is extended in the field of food.

## Materials and Methods

### Sample Information

The 2,018 samples in the experiment were collected from 2011 to 2014 in 12 regions of Southwest China. Details of the sample are shown in Supplementary Table 1. The geographical information is shown in Supplementary Figure 1; the red highlighted points are the 12 geographical areas of boletes listed in Supplementary Table 1, such as Kunming, Yuxi, and Chuxiong.

### Instruments and Reagents

An FTIR spectrometer (equipped with a DTGS detector, with a scanning range of 4,000–400 cm^–1^ and a scanning signal that accumulated 16 times with 4-cm^–1^ resolutions, PerkinElmer, United States), SY3200-T type ultrasonic cleaning instrument (with a power of 150 W and frequency of 55 kHz, Shanghai Sound Source Ultrasonic Instrument Equipment Co., Ltd., Shanghai, China), YP-2 tablet press (Shanghai Shanyue Scientific Instrument Co., Ltd., Shanghai, China), FW-100 high-speed grinder (Tianjin Huaxin Instrument Factory, Tianjin, China), and 80-μm mesh sieve (Zhejiang Shangyu Daoxu Wusi Instrument Factory, Zhejiang, China) were used.

### Mid-Infrared Spectral Acquisition

The samples were cleaned after collection, dried at a constant temperature of 50°C, crushed and passed through an 80-μm mesh sieve, and stored for later use. The 1.5 ± 0.2-mg powder of each sample and 150 ± 20-mg KBr powder were accurately weighed, then fully mixed in an agate mortar, and ground into fine powder. Finally, the fine powder was poured into the grinding tool and pressed into a sheet of uniform thickness. The FTIR spectrometer was preheated for 30 min for sample determination, and the sample was repeated two times to take the average spectrum. The interference of CO_2_ and H_2_O was deducted from the blank sample before scanning.

### Two-Dimensional Correlation Spectroscopy and Integrative Two-Dimensional Correlation Spectroscopy Spectral Acquisition

According to the theory of Noda, for the spectra measured at *m* steps with an equal interval of perturbation *t*, the dynamic spectral intensities at the variable *v* are expressed as a column vector *S* (Chen et al., 2018):


(1) $$\text{S}\left(v\right)=\left[\begin{array}{c} \begin{array}{c} \text{s}\left(v,t_{1}\right)\\ \text{s}\left(v,t_{2}\right) \end{array}\\ .\\ .\\ .\\ s\left(v,t_{m}\right) \end{array}\right]$$

A set of one-dimensional MIR spectra of *m* samples can be converted into an *m* × *n* data matrix, where *n* is the number of spectral data points. Then, the synchronous (Φ) and asynchronous (ψ) two-dimensional correlation intensities between the different frequencies *v*_1_ and *v*_2_ can be calculated as (Yang R. J. et al., 2014, 2015, 2020)


(2) $$\Phi \left(v_{1},v_{2}\right)=\frac{1}{m-1}S{\left(v_{1}\right)}^{T}\cdot S\left(v_{2}\right)$$

(3) $$\Psi \left(v_{1},v_{2}\right)=\frac{1}{m-1}S{\left(v_{1}\right)}^{T}\cdot N\cdot \text{S}\left(v_{2}\right)$$

where ***N*** is the *j-*th row and *k*-th column of the Hilbert–Noda matrix, which is defined as


(4) $$N_{jk}=\left\{ \begin{array}{c} 0,j=k\\ \frac{1}{\pi \left(k-j\right)},j\neq k \end{array}\right.$$

Synchronous 2DCOS represents the total degree of similarity between the spectral intensity measured at two different wave numbers over time. The asynchronous 2DCOS represents the difference of the dynamic change behavior of spectral intensity with time. This two-dimensional correlation analysis can be applied not only to any spectral signal that depends on the change of time but also to the spectral signal that depends on the change of other physical variables.

The integrative two-dimensional correlation intensity between the different frequencies *v*_1_ and *v*_2_ is defined as (Chen et al., 2018).


$$\text{I}\left(v_{1},v_{2}\right)=\Phi \left(v_{1},v_{2}\right)\cdot \Psi \left(v_{1},v_{2}\right)$$

(5) $$=\frac{1}{{\left(m-1\right)}^{2}}\left[S{\left(v_{1}\right)}^{T}\cdot S\left(v_{2}\right)\right]\cdot \left[S{\left(v_{1}\right)}^{T}\cdot N\cdot S\left(v_{2}\right)\right]$$

**FIGURE 1:**
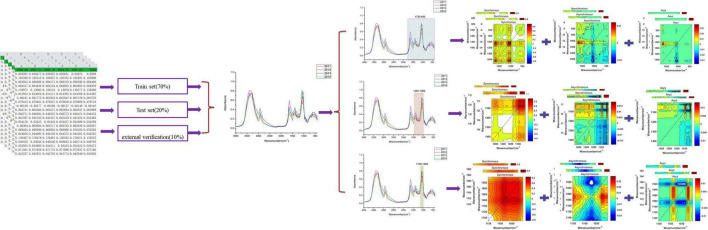
The generation processes of 2DCOS and i2DCOS.

In this article, matrix *S* (*m* × *n*) contains two spectral data (*m* = 2): the first is the average MIR spectrum and the second is the *i*-th MIR spectrum about each storage period of bolete mushrooms (Yang et al., 2013, 2015). The synchronous 2DCOS, asynchronous 2DCOS, and i2DCOS spectra of the *i*-th sample of each storage period can be obtained through Equations 2, 3, 5. We respectively intercepted 1,750– 400-, 1,450–1, 000-, and 1,150–1,000-cm^–1^ fingerprint regions and converted them into three types of 2DCOS spectra that we described above using Matlab 2017. Figure 1 shows this process.

Before deep learning modeling, we need to preprocess the 2DCOS spectral image. Firstly, we selected 90% of the samples of each storage period (1,414 samples) from all 2,018 samples for the model establishment, and the other 10% (about 200 samples) was used for external verification. Then, we used the Kennard–Stone algorithm to select the train set and test set in these 1,414 samples. After that, all one-dimensional spectral data of the train set and test set were converted into 2DCOS spectra by Matlab. Finally, we stored the 2DCOS spectra in the form of JPEG images with the same size as 128 × 128 pixels for later deep learning modeling.

The specific implementation process is shown as follows (take synchronous 2DCOS as an example):

**Step 1:** Data preparation was carried out, where data of one storage period of boletes were taken out. The data matrix is *m* × *n*, where *m* is the number of samples and *n* is the number of feature points contained in each spectrum.

**Step 2:** The Kennard–Stone algorithm was used to segment the train set (*P_train*) and test set (*P_tset*).

**Step 3:**  The chemical fingerprint data set of key feature points was obtained.

**Step 4:**  The average spectrum was calculated and stored in *mdata*.

**Step 5:** Sample = 1.

**Step 6:** A = [mdata *P_train*′ (:, sample)],

B = [mdata *P_test*′ (:, sample)],

Synchronous: Φ_train_ = n × A × A′, Φ_test_ = n × B × B′, where **$\text{n}=\frac{1}{2-1}$**;

Synchronous 2DCOS spectra were obtained by using the contour function in Matlab;

Sample = Sample + 1, if Sample ≤ m is true, continue with Step 6.

**Step 7:** If Sample ≤ m is not true, the program ends.

### Deep Learning Model Establishment

We selected MXNet as the deep learning framework, and we adopted a four-core 8G ECS cloud server which was installed with Anaconda and Python as the data processing hardware platform. Moreover, we installed MxBoard for training process visualization.

As an excellent deep learning network, ResNet could overcome the deficiency of network degradation by residual block, which is shown in Supplementary Figure 2. We can see that the residual block superimpose y = x layers (shortcut connections) on a regular shallow network whose output is *F*(*x*), so the learning objective becomes *H*(*x*) – *x*, which is the residual *F*(*x*) (He et al., 2016).

In this research, we established a 12-layer ResNet whose input data are 2DCOS and i2DCOS spectral images. In the model, we used two kinds of residual blocks. The identity residual block (identity block) is chosen to establish a model when the dimensions of input and output are consistent; the structure of the identity block is displayed in Supplementary Figure 3A. Otherwise, if the dimensions of input and output are inconsistent, we introduce the convolutional residual block (conv block) whose convolution kernel size is 1 × 1 to match the input and output dimensions (He et al., 2016). The structure of conv block is displayed in Supplementary Figure 3B.

**FIGURE 2:**
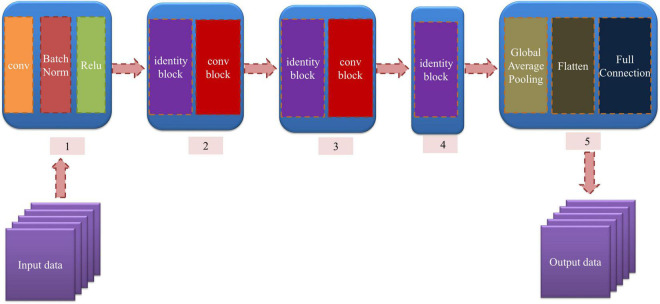
The structure of the ResNet model.

The structure of the model is displayed in Figure 2. The input data are fed into module 1, which consists of convolution, BatchNorm normalization, and Relu nonlinear activation. Then, the processed data are input into module 2, module 3, and module 4. These modules contain the identity block and conv block we mentioned above. The Global Average Pooling in module 5 is introduced to extract the important features, and the flattened layer is used to convert the multidimensional features into one dimension. Finally, the full connection layer is used for output. In our model, the weight attenuation coefficient λ of the model is 0.0001, and the learning rate is 0.01. The abscissa is the number of epochs, and the ordinate is the accuracy rate and cost function value in all the curves. The smoothing parameter of the curve was 0.6 in MxBoard.

### Deep Learning Discrimination Strategy

**FIGURE 3:**
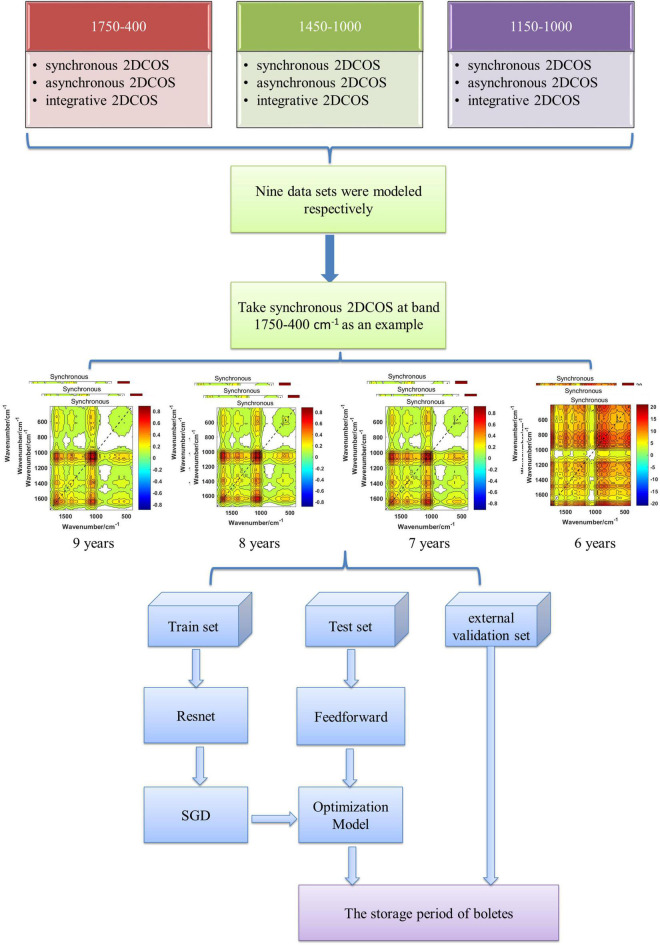
Deep learning discrimination strategy of the storage period.

The deep learning discrimination strategy for four storage periods (9, 8, 7, and 6 years) of boletes is shown in Figure 3. We acquired nine 2DCOS image datasets, which are synchronous 2DCOS, asynchronous 2DCOS, and i2DCOS spectra of 1,750– 400-, 1,450–1, 000-, and 1,150–1,000-cm^–1^ bands. A total of 18,162 2DCOS images were obtained from these nine data sets, and these digital images were modeled. With synchronous 2DCOS at band 1,750–400 cm^–1^ taken as an example, the 2DCOS images were divided into the train set, test set, and external validation set, and the details are shown in Table 1. The train set was first fed into the model for training, and then the stochastic gradient descent (SGD) method is used to find the optimal parameters and minimize the value of the loss function. The test set is used to verify whether the performance of the final selected model is optimal or not. Finally, the generalization capability of the model is verified by the external validation set.

**TABLE 1:** The details of data set partitioning.

Storage periods	The total number of samples	Train set	Test set	Validation set
		70%	20%	10%
9 years	438	307	88	43
8 years	1,034	724	207	103
7 years	254	178	51	25
6 years	292	205	58	29
Total	2,018	1,414	404	200

## Results and Discussion

### Analysis of Original Mid-Infrared Spectra

**FIGURE 4:**
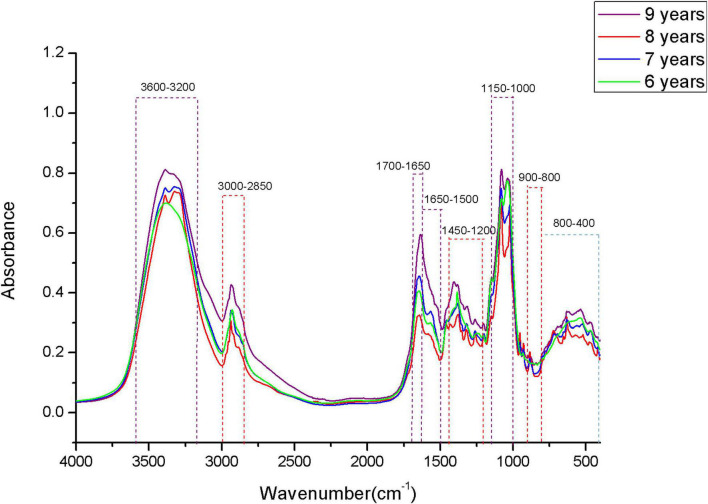
The original MIR spectra.

The original MIR spectra of four storage periods of boletes are shown in Figure 4. The band of 3,600–3,200 cm^–1^ is caused by hydroxyl stretching absorption, and this band is mainly introduced by a strong water interference, so the region is usually not selected for chemical analysis (Qi et al., 2018). The 3,000–2,850-cm^–1^ band may be methylene caused by fatty acid compounds (Fischer et al., 2006). The band of 1,700–1,650 cm^–1^ is mainly distributed by proteins, and the 1,650–1,500-cm^–1^ band is composed of amide I and amide II (Zervakis et al., 2012). Many small peaks appear in the 1,450–1,200-cm^–1^ band, which are caused by a mixture of proteins, fatty acids, and polysaccharides (Ma et al., 2016). The band of 1,000–1,200 cm^–1^ is the carbohydrate region, in which 1,032 and 1,080 cm^–1^ are considered to be chitin structures (O’Gorman et al., 2010; Mellado-Mojica and López, 2015). Glucan and mannan are mainly concentrated in the 900–800-cm^–1^ band (Ma et al., 2016). Many peaks are concentrated in the band of 900–400 cm^–1^, and the study shows that this region can be better used for chemometric analysis. It can be seen from the figure that the absorption peaks of FTIR spectra of samples in different storage periods are significantly different in each band, which indicates that the contents of various compounds may be different among samples. However, this is still difficult to distinguish by human observation, so we need to use computer methods for analysis (such as machine learning).

### The Spectral Properties of Two-Dimensional Correlation Spectroscopy

Synchronous 2DCOS are symmetric about diagonals, and the correlation peaks may appear on or outside the diagonal. The intensity peak appearing on the diagonal is called autopeak, which is the autocorrelation function value of the spectral intensity change, so the intensity of the autocorrelation peak in the 2DCOS is always positive, and its magnitude represents the total degree of dynamic change of spectral intensity in the correlation period. Therefore, in the dynamic spectrum, the regions with large intensity variations show strong autocorrelation peaks, while those regions that remain constant have minimal or no autocorrelation peaks, which indicates the influence of the microenvironment on the movement of functional groups (Noda, 1990). The cross-peaks are located outside the diagonal and represent synchronous changes in the spectral signal at different wave numbers. This synchronous variation implies that there may be paired or correlated variations in spectral intensity (Wu and Wang, 2000).

Asynchronous 2DCOS is antisymmetric about the diagonal; it has only a cross-peak but no autopeak. The different effects shown by the different ingredients of substances or chemical functional groups in the mixture can be clearly expressed in the asynchronous 2DCOS. The presence of asynchronous cross-peaks indicates that these peaks are formed from different sources or functional groups in different molecules.

It is difficult to explain the false cross-peaks with asynchronous 2DCOS due to noise interference, and the digital smoothing algorithm will reduce spectral resolution. In Equation 5, the synchronous spectrum can be considered a physical filter, while the i2DCOS can be considered as the result of the physical filtering of the asynchronous spectrum. Therefore, i2DCOS is clearer than the asynchronous spectrum (Chen et al., 2018).

### Discrimination Results of the 1,750–400-cm^–1^ Band

**FIGURE 5:**
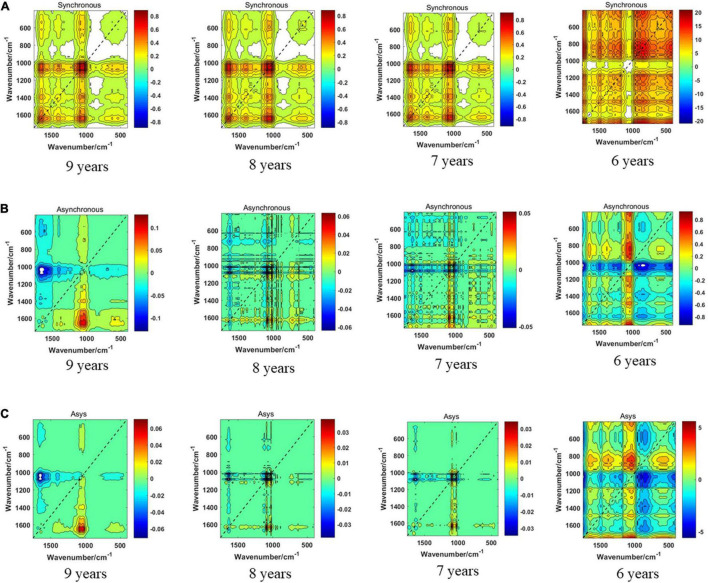
**(A)** The synchronous 2DCOS spectra of the 1,750–400-cm^–1^ band. **(B)** The asynchronous 2DCOS of the 1,750–400-cm^–1^ band. **(C)** The i2DCOS spectra of the 1,750–400-cm^–1^ band.

The synchronous 2DCOS, asynchronous 2DCOS, and i2DCOS spectra of the 1,750–400-cm^–1^ band of boletes collected from 2011 to 2014 are shown in Figure 5. In Figure 5A, the synchronous 2DCOS spectra of boletes with storage periods of 9, 8, and 7 years are similar, with a strong autopeak at 1,079 cm^–1^, which may be caused by stretching vibrations about the C–C structure of chitin in mushrooms (O’Gorman et al., 2010). There are relatively weak autopeaks at 1,403 and 1,647 cm^–1^. Among them, the autopeaks at 1,403 cm^–1^ may be due to the C–O–H bending vibration of polysaccharides and proteins (Mohacek-Grosev et al., 2001). The autopeaks at 1,647 cm^–1^ may be caused by C–N stretching vibration and C = O stretching vibration in the protein ingredients (Fischer et al., 2006). At 650 cm^–1^, there was no autopeak in the 9-year storage period, while there was a weak autopeak in the 8- and 7-year storage periods. Moreover, the cross-peaks of the three storage periods were similar. The synchronous 2DCOS spectra of the 6-year storage period are quite different from those of the other three storage periods. There is no autocorrelation peak at 1,079 cm^–1^, but there is a strong autopeak at 650 cm^–1^ and a weak autopeak at 1,200 and 1,647 cm^–1^. Figure 5B shows the asynchronous 2DCOS of boletes with different storage periods. There are two cross-peaks at 1,043 and 1,647 cm^–1^ in the spectra of the 9-year storage period. The cross-peaks in the spectra of the 8- and 7-year storage periods are very messy that it is almost impossible to get results from human observation. Similarly, the asynchronous 2DCOS spectra of the 6-year storage period are different from those of the other three storage periods, which have a lot of cross-peaks. The i2DCOS spectra of four storage periods are shown in Figure 5C. These spectra are similar to asynchronous 2DCOS, but they are much clearer. In particular, the spectra of 8- and 7-year storage periods have fewer cross-peaks.

**FIGURE 6:**
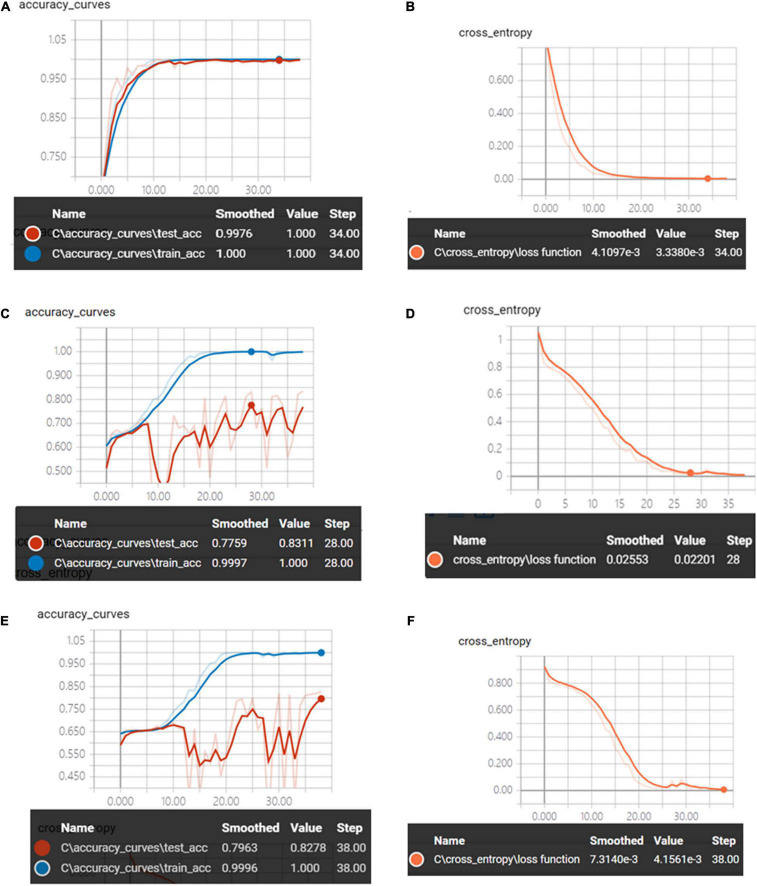
**(A)** The accuracy curves of synchronous 2DCOS spectra on the 1,750–400-cm^–1^ band. **(B)** The cross-entropy cost function of synchronous 2DCOS spectra on the 1,750–400-cm^–1^ band. **(C)** The accuracy curves of asynchronous 2DCOS spectra on the 1,750–400-cm^–1^ band. **(D)** The cross-entropy cost function of asynchronous 2DCOS spectra on the 1,750–400-cm^–1^ band. **(E)** The accuracy curves of i2DCOS spectra on the 1,750–400-cm^–1^ band. **(F)** The cross-entropy cost function of i2DCOS spectra on the 1,750–400-cm^–1^ band.

Figure 6 shows the accuracy curves and the cross-entropy cost function value of synchronous 2DCOS, asynchronous 2DCOS, and i2DCOS spectral model in the 1,750–400-cm^–1^ band. We can see that the train set accuracy of the three models is all 100%, but the accuracy of the test set is different. The test set accuracy of the synchronous 2DCOS spectral model is 100% while the loss value is 0.0033 at 34 epochs, the test set accuracy of the asynchronous 2DCOS spectral model is 83.1% while the loss value is 0.0220 at 28 epochs, and the test set accuracy of the i2DCOS spectral model is 82.3% while the loss value is 0.0042 at 38 epochs. The synchronous 2DCOS spectra have the highest accuracy, the asynchronous 2DCOS and the i2DCOS spectral models have a relatively low accuracy, and the accuracy curve fluctuates greatly, so these two models are not reliable. The loss function value of the three models is all close to zero when the epoch increases; this indicates that the models have good convergence effects.

**FIGURE 7:**
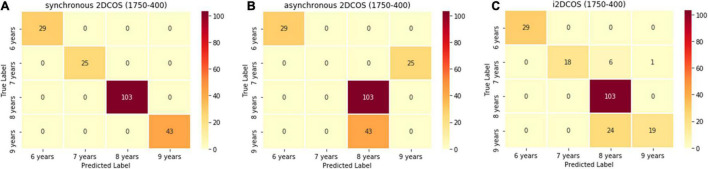
**(A)** The confusion matrix of the synchronous 2DCOS spectra model on the 1,750–400-cm^–1^ band. **(B)** The confusion matrix of the asynchronous 2DCOS spectra model on the 1,750–400-cm^–1^ band. **(C)** The confusion matrix of the i2DCOS spectra model on the 1,750–400-cm^–1^ band.

Figure 7 is the confusion matrix of the 2DCOS and i2DCOS spectral models on the 1,750–400-cm^–1^ band. There are 200 spectral images belonging to the external validation set in our study. In Figure 7A, we can see that all spectral images in the external validation set are classified correctly. It shows that the synchronous 2DCOS spectral model on the 1,750–400-cm^–1^ band has a strong generalization ability. In Figure 7B, 68 spectral images are identified incorrectly, which account for 34% of the external validation set. Among them, all the samples with a storage period of 7 years are wrongly identified as a storage period of 9 years, and all the samples with a 9-year storage period are wrongly identified as 8 years. As shown in Figure 7C, 31 samples are identified incorrectly, which account for 15.5% of the external validation set. Therefore, in terms of generalization ability on the band 1,750–400 cm^–1^, the synchronous 2DCOS model is the best, the i2DCOS model is the second best, and the asynchronous 2DCOS model is the worst.

### Discrimination Results of the 1,450–1,000-cm^–1^ Band

**FIGURE 8:**
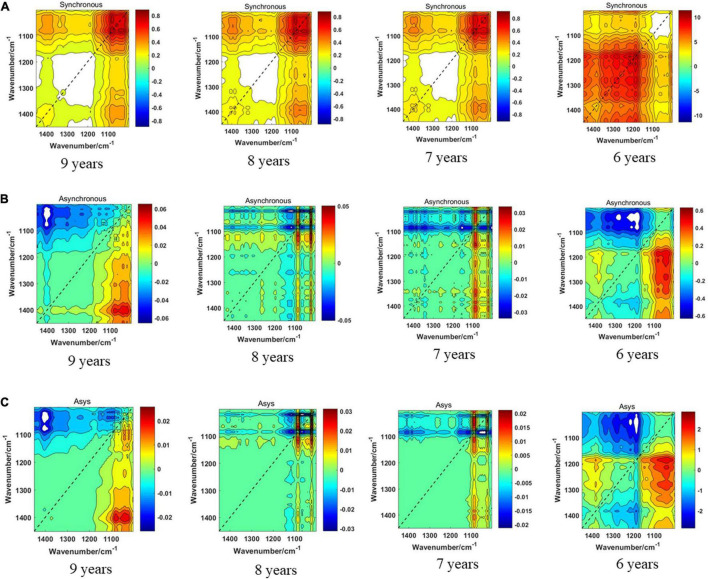
**(A)** The synchronous 2DCOS spectra of the 1,450–1,000-cm^–1^ band. **(B)** The asynchronous 2DCOS of the 1,450–1,000-cm^–1^ band. **(C)** The i2DCOS spectra of the 1,450–1,000-cm^–1^ band.

Figure 8 displays the synchronous 2DCOS, asynchronous 2DCOS, and i2DCOS spectra of four storage periods on the 1,450–1,000-cm^–1^ band of boletes. In Figure 8A, the synchronous 2DCOS of boletes with storage periods of 9, 8, and 7 years have a strong autopeak at 1,079 cm^–1^. There is a relatively weak autopeak at 1,400 cm^–1^. The cross-peaks of the three storage periods are all located at 1,400 and 1,043 cm^–1^. Similar to band 1,750–400 cm^–1^, the synchronous 2DCOS of the 6-year storage period has no autocorrelation peak at 1,079 cm^–1^, but there is a strong autopeak at 1,200 cm^–1^. In Figure 8B, the asynchronous 2DCOS of boletes with different storage periods are diverse from each other. We can see clearly that there are two cross-peaks at 1,043 and 1,403 cm^–1^ in the spectra of the 9-year storage period, and there are two cross-peaks at 1,043 and 1,200 cm^–1^ in the spectra of the 6-year storage period. The cross-peaks in the spectra of 8- and 7-year storage periods are disorganized. Figure 8C displays the i2DCOS of the four storage periods. The spectra of the 9- and 6-year storage periods have clear cross-peaks. However, the cross-peaks of the other two storage periods are not obvious.

**FIGURE 9:**
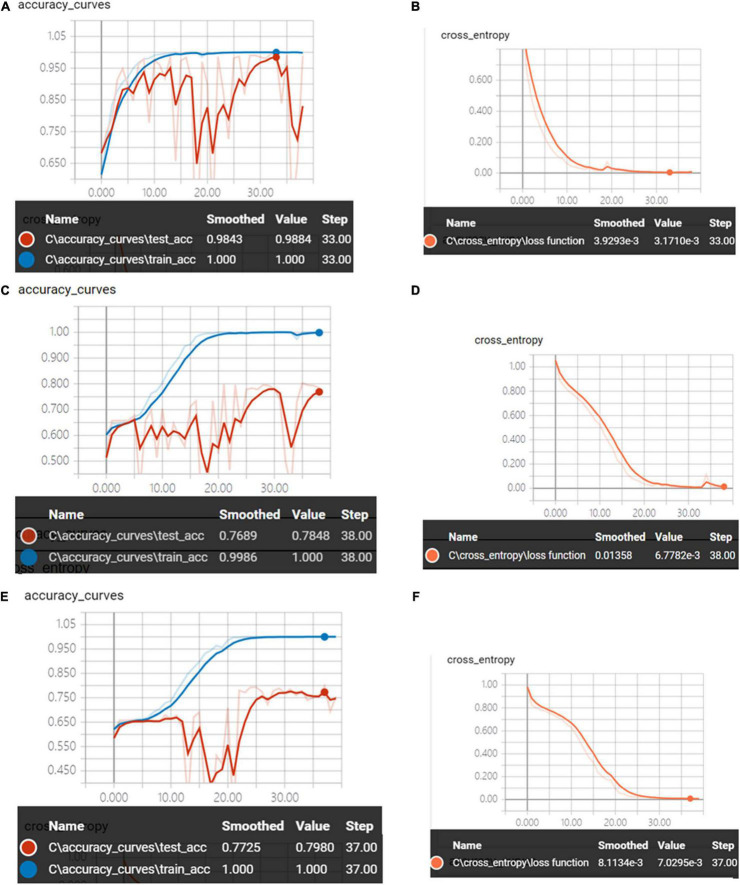
**(A)** The accuracy curves of synchronous 2DCOS spectra on 1,450–1,000-cm^–1^ band. **(B)** The cross-entropy cost function of synchronous 2DCOS spectra on the 1,450–1,000-cm^–1^ band. **(C)** The accuracy curves of asynchronous 2DCOS spectra on the 1,450–1,000-cm^–1^ band. **(D)** The cross-entropy cost function of asynchronous 2DCOS spectra on the 1,450–1,000-cm^–1^ band. **(E)** The accuracy curves of i2DCOS spectra on the 1,450–1,000-cm^–1^ band. **(F)** The cross-entropy cost function of i2DCOS spectra on the 1,450–1,000-cm^–1^ band.

Figure 9 shows the accuracy curves and the cross-entropy cost function of synchronous 2DCOS, asynchronous 2DCOS, and i2DCOS spectral models in the 1,450–1,000-cm^–1^ band. With the increase of epoch, the accuracy of the train set can reach 100%. However, the accuracy curves of the test set are not stable. The test set accuracy of synchronous 2DCOS, asynchronous 2DCOS, and i2DCOS models is up to 98.8, 78.5, and 79.8%, respectively, while the loss value is 0.0032, 0.0068, and 0.0071 at 33, 38, and 37 epochs, respectively, which are all close to zero. Similarly, the synchronous 2DCOS spectra have the highest accuracy.

**FIGURE 10:**
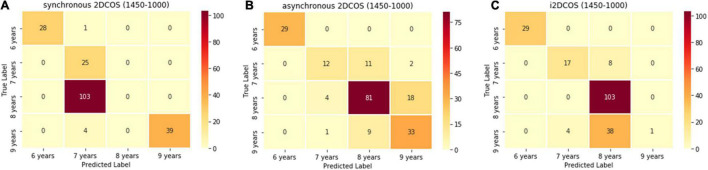
**(A)** The confusion matrix of synchronous 2DCOS spectra model on the 1,450–1,000-cm^–1^ band. **(B)** The confusion matrix of the asynchronous 2DCOS spectra model on the 1,450–1,000-cm^–1^ band. **(C)** The confusion matrix of the i2DCOS spectra model on the 1,450–1,000-cm^–1^ band.

Figure 10 is the confusion matrix of the model on the 1,750–400-cm^–1^ band. We can see from Figure 10A that the one spectral image belonging to the 6-year storage period is wrongly predicted as belonging to the 7-year storage period, the 103 spectral images belonging to the 8-year storage period are wrongly predicted as belonging to the 7-year storage period, the four spectral images belonging to the 9-year storage period are wrongly predicted as belonging to the 7-year storage period, and these account for 54% in the external validation set of the synchronous 2DCOS spectral model. Although the test set accuracy of this model is very high, its generalization ability is very poor, so it is not a good model. In Figure 10B, 45 spectral images are predicted incorrectly in the asynchronous 2DCOS spectral model, which account for 22.5% of the external validation set, and there are 50 spectral images predicted incorrectly in the i2DCOS spectral model in Figure 10C whose error rate is as high as 25%. Therefore, the generalization ability of the three models on the band 1,450–1,000 cm^–1^ is all disappointing.

### Discrimination Results of the 1,150–1,000-cm^–1^ Band

**FIGURE 11:**
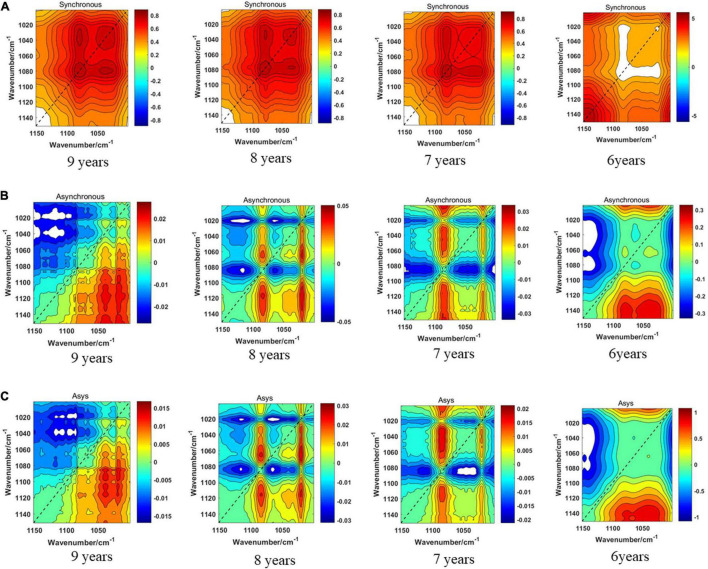
**(A)** The synchronous 2DCOS spectra of the 1,150–1,000-cm^–1^ band. **(B)** The asynchronous 2DCOS of the 1,150–1,000-cm^–1^ band. **(C)** The i2DCOS spectra of the 1,150–1,000-cm^–1^ band.

The three models of the four storage periods on the 1,150–1,000-cm^–1^ band of boletes are displayed in Figure 11. We can see from Figure 11A that the synchronous 2DCOS spectra of boletes with storage periods of 9, 8, and 7 years are almost the same. They all have a strong autopeak at 1,079 cm^–1^. Because the intercepted band is short, only one crossover peak is shown, which is magnified and clearly displayed. The 6-year storage period spectra are completely different, and there is no autocorrelation peak at 1,079 cm^–1^, so they can be recognized intuitively. Figure 11B shows the asynchronous 2DCOS of boletes with different storage periods on the 1,150–1,000-cm^–1^ band. The 9-year storage period spectra are very disordered, and computer processing is required to identify them. There are two cross-peaks at 1,025 and 1,073 cm^–1^ in the spectra of 8- and 7-year storage periods, but the intensity of the cross-peaks is different. There is no whole cross-peak in the 6-year storage period spectra. Figure 11C displays that the cross-peaks in the i2DCOS spectra of the four storage periods are the same as that of asynchronous 2DCOS on the 1,150–1,000-cm^–1^ band.

**FIGURE 12:**
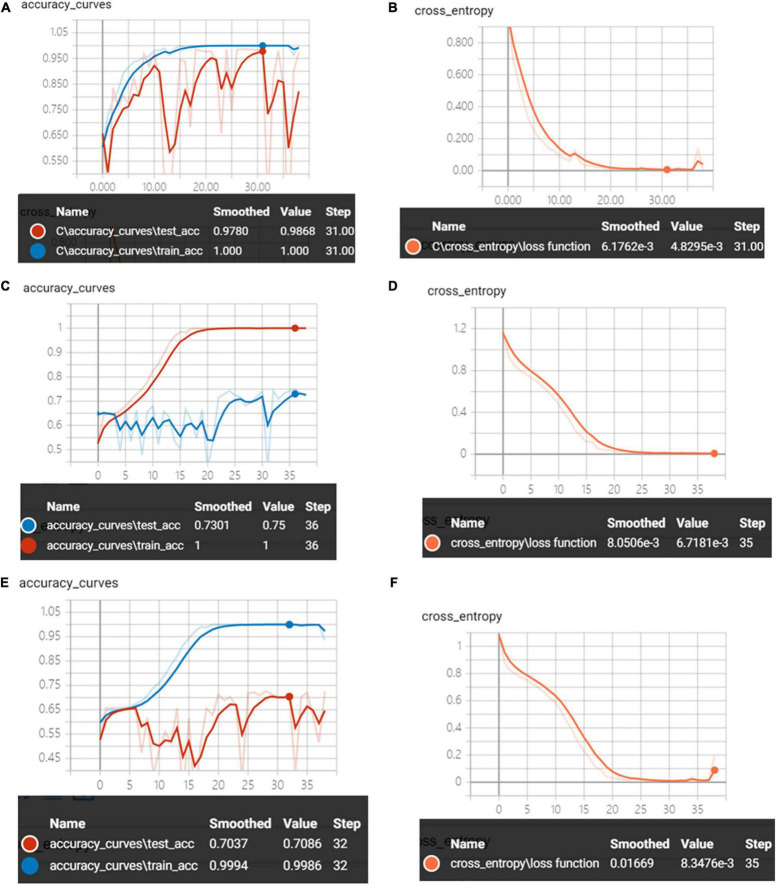
**(A)** The accuracy curves of synchronous 2DCOS spectra on the 1,150–1,000-cm^–1^ band. **(B)** The cross-entropy cost function of synchronous 2DCOS spectra on the 1,150–1,000-cm^–1^ band. **(C)** The accuracy curves of asynchronous 2DCOS spectra on the 1,150–1,000-cm^–1^ band. **(D)** The cross-entropy cost function of asynchronous 2DCOS spectra on the 1,150–1,000-cm^–1^ band. **(E)** The accuracy curves of i2DCOS spectra on the 1,150–1,000-cm^–1^ band. **(F)** The cross-entropy cost function of i2DCOS spectra on the 1,150–1,000-cm^–1^ band.

Figure 12 shows the accuracy curves and the cross-entropy cost function of synchronous 2DCOS, asynchronous 2DCOS, and i2DCOS spectral models on the 1,150–100-cm^–1^ band. We can see that the train set accuracy of synchronous 2DCOS and asynchronous 2DCOS is 100%, and the train set accuracy of the i2DCOS spectral model is 99.8%. The test set accuracy of the synchronous 2DCOS spectral model is up to 98.7% while the loss value is 0.0048 at 31 epochs, the test set accuracy of the asynchronous 2DCOS spectral model is up to 75% while the loss value is 0.0067 at 35 epochs, and the test set accuracy of the i2DCOS spectral model is up to 70.9% while the loss value is 0.0083 at 35 epochs. Though the loss function of the three models is all close to zero, the accuracy curve fluctuates greatly. Therefore, the stability of the model needs further verification.

**FIGURE 13:**
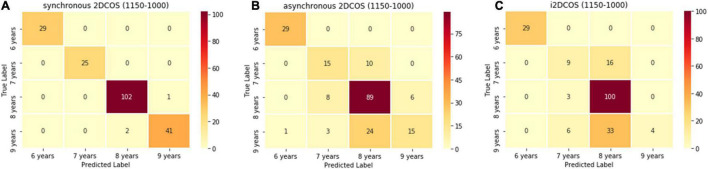
**(A)** The confusion matrix of synchronous 2DCOS spectra model on the 1,150–1,000-cm^–1^ band. **(B)** The confusion matrix of asynchronous 2DCOS spectra model on the 1,150–1,000-cm^–1^ band. **(C)** The confusion matrix of i2DCOS spectra model on the 1,150–1,000-cm^–1^ band.

The confusion matrix of the model on the 1,150–100-cm^–1^ band is shown in Figure 13. In Figure 13A, there are only three spectral images which are wrongly predicted in the synchronous 2DCOS spectral model. In Figure 13B, 52 spectral images are predicted incorrectly in the asynchronous 2DCOS spectral model, which account for 26% of the external validation set, and there are 58 spectral images predicted incorrectly in the i2DCOS spectral model in Figure 13C. The error rate of the i2DCOS spectral model is as high as 29%. In three models at the 1,150–100-cm^–1^ band, the asynchronous 2DCOS spectral model and the i2DCOS spectral model have poor generalization ability.

### Comparison of Model Recognition Results

Table 2 presents the loss value and accuracy (train set, test set, and external validation set) of nine models on bands 1,750–400, 1,450-1,000, and 1,150–1,000 cm^–1^. The model of synchronous 2DCOS on the 1,750–400-cm^–1^ band has 100% accuracy of the train set, test set and external validation set. It shows that the model has strong stability and generalization ability. The synchronous 2DCOS model on the 1,150–1,000 cm^–1^ band comes next. Its train set accuracy is 100%, test set accuracy is 98.7%, and external validation set accuracy is 98.5%. These two models have high accuracy and generalization ability, and they can be used to identify the storage period of boletes. The synchronous 2DCOS model on the 1,450–1,000-cm^–1^ band has a high accuracy rate, but its generalization ability is poor, so this model is not reliable. The asynchronous 2DCOS model and i2DCOS model on these three bands are all not good. Especially for the i2DCOS model on the 1,150–1,000-cm^–1^ band, the accuracy of the test set is only 70.9%, and the accuracy of the external verification set is only 71%. Therefore, the accuracy and generalization ability of the model are all poor. This result implies that the synchronous 2DCOS spectral model was more suitable for us to establish a deep learning model based on digital image processing in the storage period identification of boletes.

**TABLE 2:** The model comparison results.

Different bands (cm^–1^)	The type of 2DCOS	Loss value	Accuracy		
			Train set	Test set	Validation set
1,750–400	Synchronous	0.0033	100%	100%	100%
	Asynchronous	0.0054	100%	83.10%	66%
	Integrative	0.0042	100%	82.30%	84.50%
1,450–1,000	Synchronous	0.0032	100%	98.80%	46%
	Asynchronous	0.0068	100%	78.50%	77.50%
	Integrative	0.0071	100%	79.80%	75%
1,150–1,000	Synchronous	0.0048	100%	98.70%	98.50%
	Asynchronous	0.0067	100%	75%	74%
	Integrative	0.0083	99.90%	70.90%	71%

On the basis of these results, we conclude that the models of the 1,750–400-cm^–1^ band are the best, the models of the 1,150–1,000-cm^–1^ band are second best, and the models of the 1,450–1000-cm^–1^ band are relatively poor, so the band is not the key factor affecting the recognition results. Moreover, the loss values of all models are close to zero, which indicates that the loss values are not affected by band and 2DCOS types. In terms of the type of 2DCOS, the synchronous 2DCOS models are superior to asynchronous 2DCOS models and i2DCOS models. Therefore, the synchronous 2DCOS model on the 1,750–400-cm^–1^ band is the best. It may be that the color information of synchronous spectra is richer and that the line information of its autopeaks and cross-peaks are clearer on the 1,750–400-cm^–1^ band. It shows that the synchronous model of this band is more suitable for deep learning modeling.

There are a lot of surface morphological differences in different growth stages of the same species of boletes, so it is difficult to identify accurately the growth stage. In addition, fresh boletes are not easy to preserve, and its perinatal period is greatly affected by the season, so it cannot be supplied all year round and most boletes need to be dried and preserved. The smell, flavor, and nutritional value of bolete mushrooms deteriorate with the increase of storage, so the price varies significantly with the length of storage. In order to obtain greater profits, the different storage periods of bolete mushrooms in the market consider fake as real and shoddy as good. The results of this study can provide a fast, reliable, and low-cost method for the identification of the storage period of boletes, which has certain practical application value and provides a scientific basis for the quality control and market management of bolete mushrooms.

## Conclusion

In this study, 2DCOS technology combined with deep learning was used to identify the storage period of boletes in order to carry out quality control on them. We used digital image processing technology for ResNet modeling of 2DCOS spectral images, which is the innovation point of this work. By comparison, we know that the model of synchronous 2DCOS on 1,750–400- and 1,150–1,000-cm^–1^ bands has strong stability and generalization ability, and they can be used to identify the storage period of boletes. In particular, the accuracy of the test set and external validation set on the 1,750–400-cm^–1^ band can reach 100%, and its loss value is as low as 0.0033, which is close to zero. Simultaneously, the band is not the key factor affecting the recognition results, but the type of 2DCOS has a great influence on the recognition result, and the synchronous 2DCOS recognition effect is the best. In general, our method is feasible, and the identification results are reliable. In the following research, we need to continue to expand the number of samples and study new deep learning models. Of course, we need to generalize the current model to other applications.

## Data Availability Statement

The raw data supporting the conclusions of this article will be made available by the authors, without undue reservation.

## Author Contributions

J-ED: conceptualization, methodology, formal analysis, and writing – original draft. JZ: formal analysis and data curation. TL: project administration. Y-ZW: resources and supervision. All authors contributed to the article and approved the submitted version.

## Conflict of Interest

The authors declare that the research was conducted in the absence of any commercial or financial relationships that could be construed as a potential conflict of interest.

## Publisher’s Note

All claims expressed in this article are solely those of the authors and do not necessarily represent those of their affiliated organizations, or those of the publisher, the editors and the reviewers. Any product that may be evaluated in this article, or claim that may be made by its manufacturer, is not guaranteed or endorsed by the publisher.
