# Supplementary material for: The Storage Period Discrimination of Bolete Mushrooms Based on Deep Learning Methods Combined With Two-Dimensional Correlation Spectroscopy and Integrative Two-Dimensional Correlation Spectroscopy
Source: Front Microbiol. 2021 Nov 25;12:771428. doi: 10.3389/fmicb.2021.771428 (PMC8656461; doi:10.3389/fmicb.2021.771428)
Supplement: Supplementary file 1 [file Data_Sheet_1.docx]

Supplementary Material

# Supplementary Data

# There is no supplementary data in this paper.

# Supplementary Figures and Tables

# Supplementary Figures and Tables caption：

Fig. s1 The geographical information of the samples

# Fig.s2 Schematic diagram of residual block

# Fig.s3 (A) The identity block (B) The conv block

# Table s1 The detail information of the samples

## Supplementary Figures


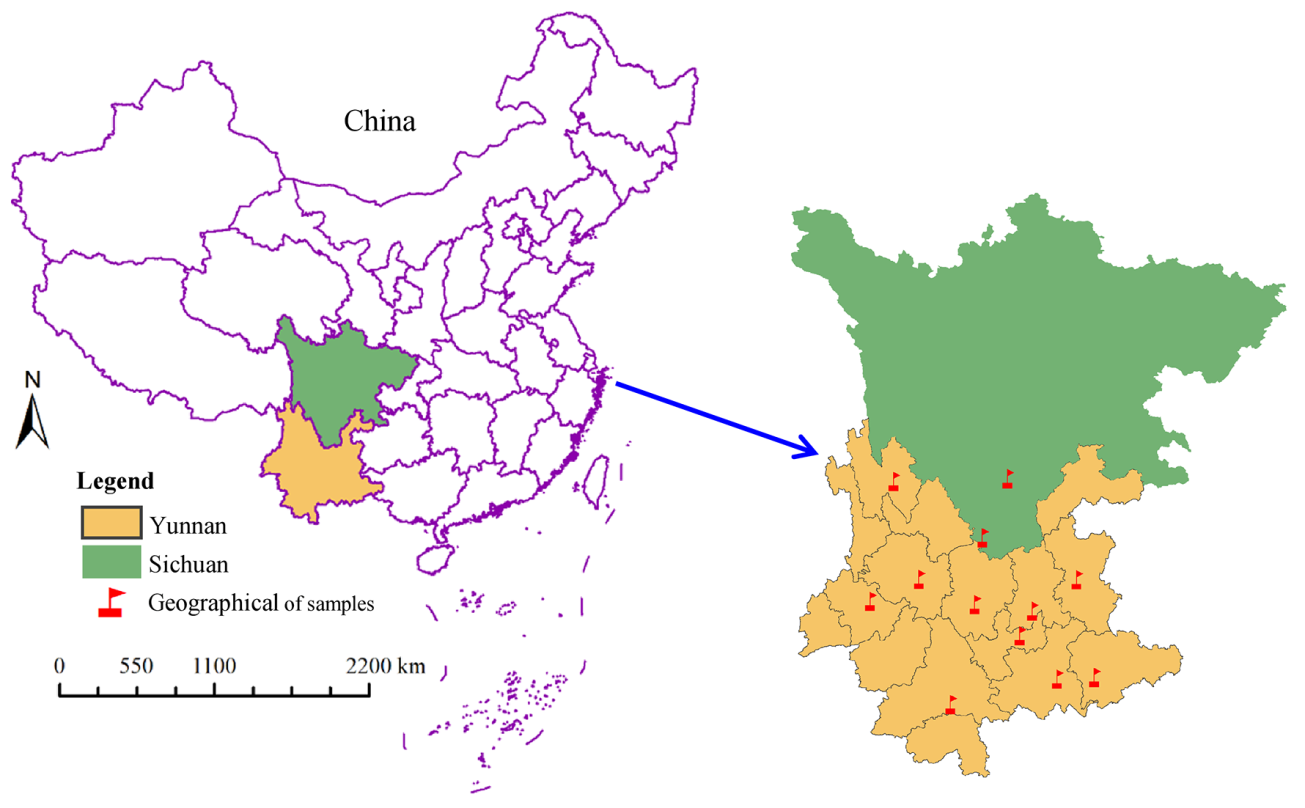


## Fig.s1 The geographical information of the samples


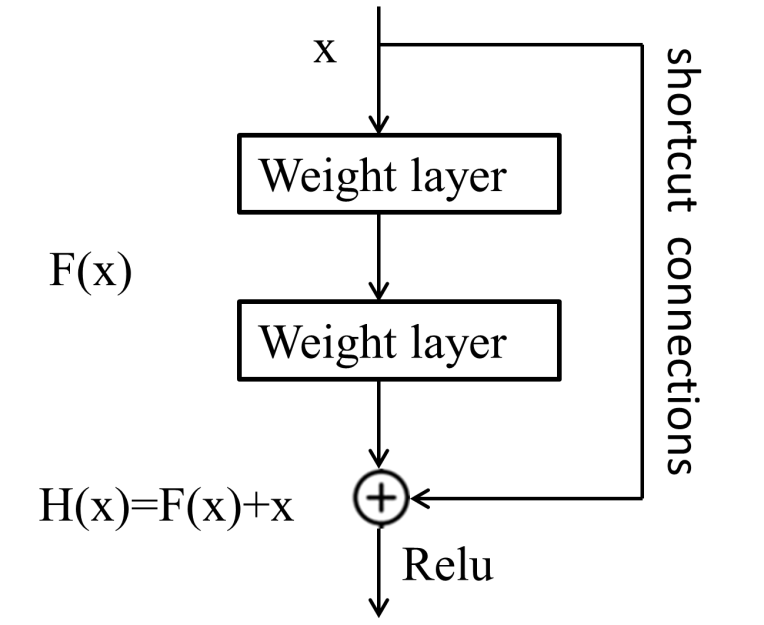


**Fig.s2** Schematic diagram of residual block


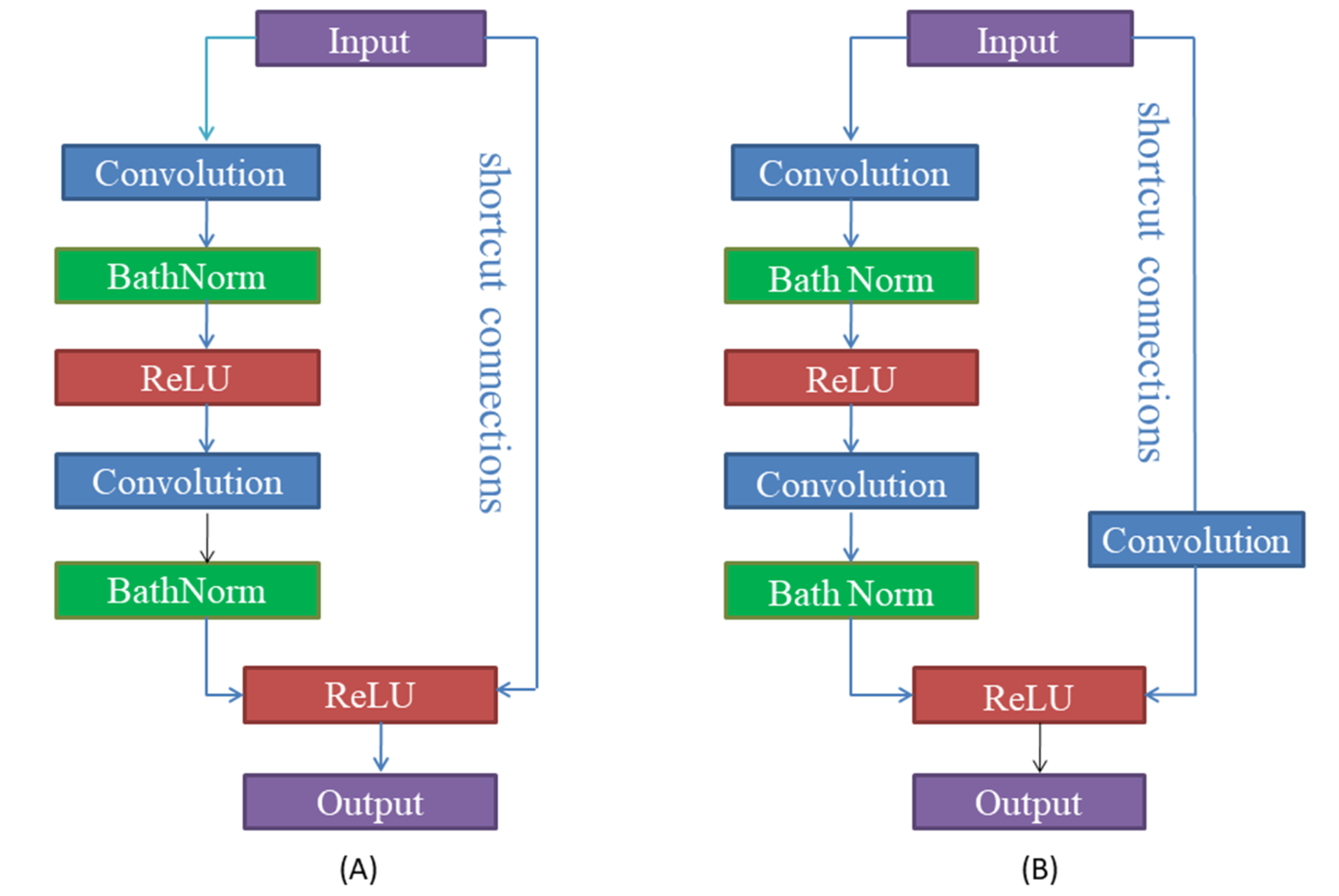


**Fig.s3** (A) The identity block (B) The conv block

## Supplementary Tables

**Table s1 The detail information of the samples**

| **Storage period** | **Acquisition time** | **Species** | **Sampling position** | **Number of fruiting body** |
| --- | --- | --- | --- | --- |
| 9 years | 2011 | *Harrya chromipes (Frost) Halling, Nuhn, Osmundson & Manfr. Binder* | Fumin,Kunming | 20 |
|  |  | *Harrya chromipes (Frost) Halling, Nuhn, Osmundson &Manfr. Binder* | Wuhuaqu,Kunming | 20 |
|  |  | *Retiboletus griseus (Frost) Manfr. Binder & Bresinsky* | Eshan,Yuxi | 20 |
|  |  | *Retiboletus griseus (Frost) Manfr. Binder & Bresinsky* | Wuhuaqu,Kunming | 16 |
|  |  | *Leccinellum griseum (Quél.) Bresinsky & Manfr. Binder* | Wuhuaqu,Kunming | 14 |
|  |  | *Leccinellum griseum (Quél.) Bresinsky & Manfr. Binder* | Wuding,Chuxiong | 20 |
|  |  | *Boletus umbriniporus Hongo* | Yimen,Yuxi | 20 |
|  |  | *Boletus bainiugan Dentinger* | Yimen,Yuxi | 22 |
|  |  | *Boletus bainiugan Dentinger* | Nanhualongchuan,Chuxiong | 20 |
|  |  | *Boletus bainiugan Dentinger* | Nanhuashaqiao,Chuxiong | 16 |
|  |  | *Boletus tomentipes Earle* | Eshanfuliangpeng,Yuxi | 18 |
|  |  | *Boletus tomentipes Earle* | Eshanxiaojie,Yuxi | 18 |
|  |  | *Boletus tomentipes Earle* | Yaoan,Chuxiong | 14 |
|  |  | *Boletus tomentipes Earle* | Nanhua,Chuxiong | 14 |
|  |  | *Boletus tomentipes Earle* | Nanbanghe,Pu'er | 21 |
|  |  | *Boletus tomentipes Earle* | Zezhou,Qujing | 14 |
|  |  | *Boletus bicolor Raddi* | Nanhua,Chuxiong | 13 |
|  |  | *Boletus bicolor Raddi* | Cangshan,Dali | 14 |
|  |  | *Boletus bicolor Raddi* | Zezhou,Qujing | 14 |
|  |  | *Boletus speciosus Forst.* | Nanhua,Chuxiong | 14 |
|  |  | *Leccinum rugosiceps (Peck) Singer* | Wuhuaqu,Kunming | 16 |
|  |  | *Leccinum rugosiceps (Peck) Singer* | Zezhou,Qujing | 20 |
|  |  | *Leccinum rugosiceps (Peck) Singer* | Yimen,Yuxi | 20 |
|  |  | *Leccinum rugosiceps (Peck) Singer* | Simao,Pu'er | 20 |
|  |  | *Leccinum rugosiceps (Peck) Singer* | Fumin,Kunming | 20 |
| 8 years | 2012 | *Harrya chromipes (Frost) Halling, Nuhn, Osmundson &Manfr. Binder* | Jiuxi,Yuxi | 14 |
|  |  | *Sutorius magnificus (W.F. Chiu) G. Wu & Zhu L. Yang* | Jiangchuan,Yuxi | 20 |
|  |  | *Sutorius magnificus (W.F. Chiu) G. Wu & Zhu L. Yang* | Yimentongchang,Yxi | 20 |
|  |  | *Sutorius magnificus (W.F. Chiu) G. Wu & Zhu L. Yang* | Gejiu,Honghe | 14 |
|  |  | *Sutorius magnificus (W.F. Chiu) G. Wu & Zhu L. Yang* | Yimenjiangkou,Yuxi | 14 |
|  |  | *Sutorius magnificus (W.F. Chiu) G. Wu & Zhu L. Yang* | Midu,Dali | 18 |
|  |  | *Sutorius magnificus (W.F. Chiu) G. Wu & Zhu L. Yang* | Simao,Pu'er | 20 |
|  |  | *Retiboletus griseus (Frost) Manfr. Binder & Bresinsky* | Longyangqu,Baoshan | 20 |
|  |  | *Retiboletus griseus (Frost) Manfr. Binder & Bresinsky* | Jiangchuan,Yuxi | 19 |
|  |  | *Retiboletus griseus (Frost) Manfr. Binder & Bresinsky* | Midu,Dali | 19 |
|  |  | *Retiboletus griseus (Frost) Manfr. Binder & Bresinsky* | Jinning,Kunming | 14 |
|  |  | *Retiboletus griseus (Frost) Manfr. Binder & Bresinsky* | Malong,Qujing | 14 |
|  |  | *Retiboletus griseus (Frost) Manfr. Binder & Bresinsky* | Anning,Kunming | 14 |
|  |  | *Retiboletus griseus (Frost) Manfr. Binder & Bresinsky* | Shilin,Kunming | 20 |
|  |  | *Boletus umbriniporus Hongo* | Longyangqu,Baoshan | 20 |
|  |  | *Boletus umbriniporus Hongo* | Midu,Dali | 16 |
|  |  | *Boletus umbriniporus Hongo* | Shiping,Honghe | 18 |
|  |  | *Boletus umbriniporus Hongo* | Yuanmou,Chuxiong | 20 |
|  |  | *Boletus umbriniporus Hongo* | Huangcaoba,Yuxi | 16 |
|  |  | *Boletus umbriniporus Hongo* | Yimen,Yuxi | 20 |
|  |  | *Boletus umbriniporus Hongo* | Gejiu,Honghe | 20 |
|  |  | *Boletus bainiugan Dentinger* | Pudacuo,Diqing | 20 |
|  |  | *Boletus bainiugan Dentinger* | Midu,Dali | 20 |
|  |  | *Boletus bainiugan Dentinger* | Weixi,Diqing | 14 |
|  |  | *Boletus bainiugan Dentinger* | Longyangqu,Baoshan | 20 |
|  |  | *Boletus bainiugan Dentinger* | Anningwenshui,Kunming | 20 |
|  |  | *Boletus bainiugan Dentinger* | Anningfengyi,Kunming | 20 |
|  |  | *Boletus bainiugan Dentinger* | Yimen,Yuxi | 54 |
|  |  | *Boletus bainiugan Dentinger* | Heqing,Dali | 14 |
|  |  | *Boletus bainiugan Dentinger* | Dongshan,Wenshan | 14 |
|  |  | *Boletus bainiugan Dentinger* | Anningbajie,kunming | 16 |
|  |  | *Boletus bainiugan Dentinger* | Shilin,Kunming | 20 |
|  |  | *Boletus bainiugan Dentinger* | Malongjiuxian,Qujing | 14 |
|  |  | *Boletus tomentipes Earle* | Gejiu,Honghe | 14 |
|  |  | *Boletus tomentipes Earle* | Shangri-la,Diqing | 17 |
|  |  | *Boletus tomentipes Earle* | Yimen,Yuxi | 20 |
|  |  | *Boletus tomentipes Earle* | Eshanchahe,Yuxi | 14 |
|  |  | *Boletus tomentipes Earle* | Shiping,Honghe | 16 |
|  |  | *Boletus tomentipes Earle* | Dechang,Liangshan | 18 |
|  |  | *Boletus tomentipes Earle* | Heqing,Dali | 14 |
|  |  | *Boletus tomentipes Earle* | Miyi,Panzhihua | 20 |
|  |  | *Boletus bicolor Raddi* | Jiangchuan,Yuxi | 20 |
|  |  | *Retiboletus griseus (Frost) Manfr. Binder & Bresinsky* | Yongren,Chuxiong | 20 |
|  |  | *Retiboletus griseus (Frost) Manfr. Binder & Bresinsky* | Dongshan,Wenshan | 14 |
|  |  | *Boletus speciosus Forst.* | Yimen,Yuxi | 20 |
|  |  | *Boletus speciosus Forst.* | Anning,Kunming | 20 |
|  |  | *Boletus speciosus Forst.* | Longyangqu,Baoshan | 20 |
|  |  | *Boletus speciosus Forst.* | Yuanmou,Chuxiong | 20 |
|  |  | *Boletus speciosus Forst.* | Anning,Kunming | 20 |
|  |  | *Leccinum rugosiceps (Peck) Singer* | Shiping,Honghe | 19 |
|  |  | *Leccinum rugosiceps (Peck) Singer* | Midu,Dali | 20 |
|  |  | *Leccinum rugosiceps (Peck) Singer* | Anning,Kunming | 20 |
|  |  | *Leccinum rugosiceps (Peck) Singer* | Weixi,Diqing | 14 |
|  |  | *Leccinum rugosiceps (Peck) Singer* | Shilin,Kunming | 16 |
|  |  | *Leccinum rugosiceps (Peck) Singer* | Longyangqu,Baoshan | 14 |
|  |  | *Leccinum rugosiceps (Peck) Singer* | Yuanmou,Chuxiong | 14 |
|  |  | *Leccinum rugosiceps (Peck) Singer* | Yimen,Yuxi | 14 |
| 7 years | 2013 | *Retiboletus griseus (Frost) Manfr. Binder & Bresinsky* | Lufeng,Chuxiong | 20 |
|  |  | *Tylopilus felleus (Bull.) P. Karst.* | Hongtaqu,Yuxi | 10 |
|  |  | *Tylopilus felleus (Bull.) P. Karst.* | Zezhou,Qujing | 8 |
|  |  | *Boletus umbriniporus Hongo* | Hongtaqu,Yuxi | 2 |
|  |  | *Boletus umbriniporus Hongo* | Zezhou,Qujing | 10 |
|  |  | *Boletus umbriniporus Hongo* | Nanhua,Chuxiong | 16 |
|  |  | *Boletus bainiugan Dentinger* | Malong,Qujing | 16 |
|  |  | *Boletus bainiugan Dentinger* | Zezhou,Qujing | 8 |
|  |  | *Boletus bainiugan Dentinger* | Nanhua,Chuxiong | 16 |
|  |  | *Boletus bainiugan Dentinger* | Dayingjie,Yuxi | 16 |
|  |  | *Boletus bicolor Raddi* | Dayingjie,Yuxi | 20 |
|  |  | *Boletus speciosus Forst.* | Zezhou,Qujing | 10 |
|  |  | *Boletus speciosus Forst.* | Dayingjie,Yuxi | 16 |
|  |  | *Heimioporus retisporus (Pat. & C.F. Baker) E. Horak* | Lufeng,Chuxiong | 20 |
|  |  | *Rugiboletus extremiorientalis (Lj.N. Vassiljeva) G. Wu & Zhu L. Yang* | Hongtaqu,Yuxi | 2 |
|  |  | *Rugiboletus extremiorientalis (Lj.N. Vassiljeva) G. Wu & Zhu L. Yang* | Nanhua,Chuxiong | 8 |
|  |  | *Rugiboletus extremiorientalis (Lj.N. Vassiljeva) G. Wu & Zhu L. Yang* | Jiulongchi,Yuxi | 16 |
|  |  | *Rugiboletus extremiorientalis (Lj.N. Vassiljeva) G. Wu & Zhu L. Yang* | Caoba,Yuxi | 20 |
|  |  | *Rubroboletus sinicus (W.F. Chiu) Kuan Zhao & Zhu L. Yang* | Malong,Qujing | 6 |
|  |  | *Rubroboletus sinicus (W.F. Chiu) Kuan Zhao & Zhu L. Yang* | Jiulongchi,Yuxi | 14 |
| 6 years | 2014 | *Suillus spraguei (Berk. & M.A. Curtis) Kuntze* | Zhenyuan,Pu'er | 9 |
|  |  | *Suillus spraguei (Berk. & M.A. Curtis) Kuntze* | Jiuxi,Yuxi | 14 |
|  |  | *Sutorius magnificus (W.F. Chiu) G. Wu & Zhu L. Yang* | Dayingjie,Yuxi | 14 |
|  |  | *Retiboletus griseus (Frost) Manfr. Binder & Bresinsky* | Zhenyuan,Pu'er | 12 |
|  |  | *Retiboletus griseus (Frost) Manfr. Binder & Bresinsky* | Dayingjie,Yuxi | 14 |
|  |  | *Boletus bainiugan Dentinger* | Zhenyuan,Pu'er | 7 |
|  |  | *Boletus bainiugan Dentinger* | Xinping,Yuxi | 14 |
|  |  | *Boletus bainiugan Dentinger* | Jiulongchi,Yuxi | 20 |
|  |  | *Boletus bainiugan Dentinger* | Dayingjie,Yuxi | 16 |
|  |  | *Boletus tomentipes Earle* | Xinping,Yuxi | 20 |
|  |  | *Boletus tomentipes Earle* | Eshanshuangjiang,Yuxi | 12 |
|  |  | *Boletus tomentipes Earle* | Dayingjie,Yuxi | 18 |
|  |  | *Boletus bicolor Raddi* | Zhenyuan,Pu'er | 8 |
|  |  | *Boletus bicolor Raddi* | Dayingjie,Yuxi | 12 |
|  |  | *Retiboletus griseus (Frost) Manfr. Binder & Bresinsky* | Xinping,Yuxi | 4 |
|  |  | *Boletus speciosus Forst.* | Eshan,Yuxi | 20 |
|  |  | *Boletus speciosus Forst.* | Beicheng,Yuxi | 20 |
|  |  | *Gyroporus ballouii (Peck) E. Horak* | Jiuxi,Yuxi | 16 |
|  |  | *Rugiboletus extremiorientalis (Lj.N. Vassiljeva) G. Wu & Zhu L. Yang* | Zhenyuan,Pu'er | 6 |
|  |  | *Rugiboletus extremiorientalis (Lj.N. Vassiljeva) G. Wu & Zhu L. Yang* | Eshan,Yuxi | 16 |
|  |  | *Boletus ferrugineus Schaeff.* | Dayingjie,Yuxi | 20 |
